# Notes and News

**Published:** 1900-05-05

**Authors:** 


### Notes and News.

The Paris Academy of Medicine have declared it advisable that infectious pneumonia and broncho-pneumonia be added to the list of diseases which should be compulsorily notified.

Mr. Treves' address on Military Surgery in South Africa, to be given before the Royal Medical and Chirurgical Society on Tuesday, May 8th, will elicit considerable interest.

The men belonging to the medical service of the German Army must in future learn to ride the bicycle. They are to be able to use it either on the march or in cantonments. This seems a highly reasonable arrangement.

The King and Queen of Italy and also the Crown Prince and Princess of Naples attended the opening of the International Congress on Tuberculosis at Naples on April 25th. The delegates of most of the Governments of European countries were present, besides other notabilities, scientific and social.

Professor Dewar delivered the first of a course of lectures on "A Century of Chemistry" before the Royal Institute last week. The discourse bore principally upon the work of Sir Humphry Davy, and the lecturer repeated some of Davy's experiments with his own instruments, showing that the deductions then drawn had been confirmed by subsequent tests.

The horrors of cholera are added to those of famine and plague in some parts of India. From Bombay we learn that cholera is raging at the great camp at Godhra, which contains thousands of natives who are receiving famine relief from the Government.

A new fireman's device, recently introduced into the London Fire Brigade, is the "smoke cap," a helmet enclosing the face something like that used by divers. Into this air is constantly pumped through a long line of piping, so that it is possible by aid of this apparatus to penetrate far into buildings filled with smoke of such density as to be absolutely fatal if respired. An apparatus of this kind has long been advocated for use in chlorine works.

It is reported that, added to the hardship which the American soldiers are undergoing in the Philippines, they are suffering terribly from the tropical diseases to which they are exposed.

A new cottage hospital was opened last week at Ramsbottom, in Lancashire. The new building, which contains eight beds and a private ward for paying patients, has been presented to the town by Mr. and Mrs. Aitken, of Holcombe Hall.

A three days' conference on medical organisation has been held at Manchester this week, under the auspices of the Medical Guild. Mr. G. H. Wright, F.R.C.S., acted as president of the conference, which was opened by Alderman Walmsley, ex-Mayor of Salford.

Mrs. Beerbohm Tree has sent 50 guineas to the Prince of Wales's Hospital Fund, as a thank-offering with regard to His Royal Highness's recent happy escape. She suggests that others should contribute to the Fund to commemorate in a lasting manner the nation's rejoicing.

The annual dinner of the students of King's College, London, will be held at the Holborn Restaurant, on Monday, June 18th, with the Hon. Sir John Alexander Cockburn, K.C.M.G., F. and A.K.C., M.D., Agent-General for, and formerly Premier of, South Australia, in the chair. The Duke of Cambridge has signified his intention of being present.

Very serious views of the plague are taken in Sydney, and the well-to-do classes are leaving the town in large numbers. The last report of cases, however, does not show an increase. The New South Wales Cabinet are about to take possession of the foreshores of Darling Harbour, in the vicinity of which most of the cases have appeared. They will now be able to take every precautionary measure. The appearance of cases at Port Said is causing anxiety, and the number of places where cases of plague are present is now very large.

Mr. A. Jones, of Liverpool, has, in connection with the Liverpool School of Tropical Medicine, set on foot a scheme to assist natives of our colonies in the Tropics to study at the school. The African Steamship Company is co-operating in the movement. It is proposed that students should pursue the whole course of medical education at Liverpool, and take a degree there. It is calculated that the whole expense of the five years' period of study would amount to £600.

The German Congress of Medicine held its annual meeting at Wiesbaden, commencing April 8th. Professor von Jaksh, of Prague, opened the congress. The first subject under consideration was "Pneumonia and Its Treatment," opened by Professor von Koranyi, of Buda Pesth. Mediterannean Fever, Gouty Affections, Influenza and Chronic Heart Disease, Digestive Disturbances, Albumen in the Diabetic Diet, New Methods of Ascertaining the Cardiac Boundaries, and many other subjects formed a programme of the most varied and comprehensive description.

